# A scaling aneurysm model-based approach to assessing the role of flow pattern and energy loss in aneurysm rupture prediction

**DOI:** 10.1186/s12967-015-0673-z

**Published:** 2015-09-22

**Authors:** Yunling Long, Jingru Zhong, Hongyu Yu, Huagang Yan, Zhizheng Zhuo, Qianqian Meng, Xinjian Yang, Haiyun Li

**Affiliations:** Department of Biomedical Engineering, School of Biomedical Engineering, Capital Medical University, Fengtai District, Beijing, 100069 China; Beijing Neurosurgical Institute, Beijing Tiantan Hospital, Beijing, 100050 China

**Keywords:** Intracranial aneurysm, Aspect ratio, Energy loss, Fluid–structure interaction, Flow pattern

## Abstract

**Background and purpose:**

Energy loss (EL) was regarded to be one of the key parameters in predicting the rupture risk of IA. In this paper, we took varied aspect ratio (AR) as a scaling law to create a series of longitudinal models to investigate the longitudinal changes of flow pattern and EL as the AR varies, in order to explore the relationship between the longitudinal characteristic EL parameters with aneurysm rupture risk.

**Methods:**

Seven original intracranial aneurysms (IA) models with similar locations were reconstructed from patient 3D rotational angiography (3DRA) images. Based on these models, a series of scaling aneurysm models with different ARs were created with our proposed scaling algorithms. Fluid–solid interaction (FSI) simulations were performed on every model to obtain hemodynamics flow pattern and EL.

**Results:**

With AR increasing, flow pattern became more complex, with vortices appearing gradually in the aneurysms (AR > 1.5). Furthermore, the velocity significantly decreased in aneurysms with high ARs (>1.5). Meanwhile, the aneurysm EL increased with increasing AR. Once AR exceeded 1.5, EL changed drastically.

**Conclusion:**

EL was a potential parameter predicting future rupture of unruptured aneurysms. If the EL during the growth of the unruptured aneurysms increased sharply, we strongly recommend an intervention.

## Background

Intracranial aneurysm is a localized dilation of cerebral vascular wall [1–3]. Ruptured cerebral aneurysm leads to subarachnoid hemorrhage, accompanied by high mortality and morbidity rate [4–7]. With the development of minimally invasive neuroimaging technologies, unruptured aneurysms are frequently detected. On the other hand, preventive treatment of unruptured intracranial aneurysms has become a controversy [8, 9]. It is believed that the rupture risk of some unruptured intracranial aneurysms in the future is lower than the risk of complications (morbidity and mortality) caused by prophylactic treatment. Consequently, it is worthwhile to rupture risk assessment of unruptured aneurysms in clinic. Some morphologic and hemodynamic factors, such as aneurysm size, aspect ratio, undulation index, concentrated flow impingement zones, and complex flow patterns, are predictors of the rupture risk of cerebral aneurysms. Among them, aspect ratio—IA perpendicular height/neck diameter, abbreviated AR—has been found to be statistically important [10]. Aneurysms with higher AR posed higher rupture risks [11–13]. Furthermore, complex flow patterns have been thought to increase inflammatory cell infiltration in the aneurysmal wall, thereby increasing rupture risk [14]. Cebral et al. [1] demonstrated that 72 % of ruptured aneurysms had complex flow patterns, 80 % had possessed small impingement regions, and 76 % had small jet sizes; Castro et al. [15] studied 26 aneurysms and found that aneurysms with small impaction zones were more prone to rupture than those with large ones. In addition, the hemodynamic EL is also deemed to be one of the key parameters in predicting the rupture risk of IA. In recent years, several studies have investigated the relationship between EL and rupture risk of IA. Qian Y et al. [16] studied the EL difference between 4 ruptured and 26 unruptured IAs situated at ICA–posterior communicating artery and found that the EL of ruptured IAs was 5 times as high as those of unruptured stable ones (ruptured, 0.00374 ± 0.0011; stable, 0.000745 ± 0.0001 mW/mm^3^, P < 0.001). Although Qian et al. showed that the ruptured aneurysms had higher energy loss, they had only four ruptured aneurysms in their study, two behaving similar to unruptured aneurysms. Meanwhile, Cebral et al. [17] found that ruptured aneurysms had high kinetic energy ratio (not significant), but lower viscous dissipation than unruptured aneurysms. Takao et al. [18] investigated the EL of 50 side-wall internal carotid posterior communicating artery aneurysms and 50 middle cerebral artery bifurcation aneurysms and found that energy loss showed a higher, but statistically not significant, tendency in ruptured aneurysms. These studies did not show a consistent conclusion. We speculated that the inconsistent results were mainly caused by difference in the parent artery of these studied aneurysm models and not enough longitudinal IA morphologies are available for the follow-up study due to the high risk of IA rupture. Previous studies were used to focus on horizontal analysis.

Previous studies have centered on horizontal risk analysis based on the rupture or unrupture aneurysm samples acquired at a time. These studies have already obtained the geometrical parameters and hemodynamic parameters associated with the rupture risk of aneurysm. However, these parameters are horizontal state parameters, which are independent of the process. In this paper, we took varied AR as a scaling factor to create a series of longitudinal models to eliminate the influence of load tumor blood vessels and explored the variations of flow pattern and EL in longitudinal models, in order to further perform the longitudinal risk assessment of unruptured aneurysm. The longitudinal prediction analysis based on the method was applied to obtain the longitudinal course characteristic parameters of aneurysm rupture risk. Analysis of the longitudinal course characteristic parameters of aneurysm is helpful to explore the hemodynamic mechanism of IAs rupture, and it is helpful for the clinical diagnosis and evaluation of the development of aneurysms.

## Methods

### Patients and imaging data

Seven unruptured cerebral aneurysms situated on internal carotid posterior communicating artery were virtually reconstructed as original models from patient 3DRA images. The patient-specific 3DRA data were acquired from a Philips Allura Xper Digital Subtraction system (Allura Xper; Philips Medical System). The C-arm of the Digital Subtraction system rotated twice. The first rotation produced the subtraction mask. The second rotation was carried out simultaneously with the administration of contrast agent. The projection images were transferred to the Integral 3DRA Workstation (Philips Healthcare) and reconstructed into 3D voxel data by using standard proprietary software (XtraVision, Philips Healthcare). The consent of the patient was secured for using the data. This study was approved by the Ethics Committee of Beijing Neurosurgical Institute.

### The scaling models

Our proposed aneurysm scaling models were built by resizing the aneurysm sacs of the original IA models and keeping other morphological parameters constant. The aneurysm neck cutting plane, where the physician put a clip during craniotomy, was determined by an experienced physician. The cutting plane was used to separate the aneurysm from the parent vessel. Once the IA sac was resized, the scaled IA sac and parent vessels were saved as integrated data in stereolithography (STL) file format for later FSI analysis. The exact mathematical formulae for resizing the aneurismal sacs of the original models are presented as follows:

1. A new coordinate system (X, Y, Z) was established, and a rigid transformation formula was applied to make the aneurysmal neck plane parallel to the XY plane in the new coordinate system:
  1 $$\left[ {\begin{array}{*{20}c} x \\ y \\ z \\ 1 \\ \end{array} } \right] = \left[ {\begin{array}{*{20}c} 1 & 0 & 0 & p \\ 0 & 1 & 0 & q \\ 0 & 0 & 1 & r \\ 0 & 0 & 0 & 1 \\ \end{array} } \right] \times \left[ {\begin{array}{*{20}c} {\cos \varPhi } & {\sin \varPhi } & 0 & 0 \\ { - \sin \varPhi } & {\cos \varPhi } & 0 & 0 \\ 0 & 0 & 1 & 0 \\ 0 & 0 & 0 & 1 \\ \end{array} } \right] \times \left[ {\begin{array}{*{20}c} {\cos \omega } & 0 & { - \sin \omega } & 0 \\ 0 & 1 & 0 & 0 \\ {\sin \omega } & 0 & {\cos \omega } & 0 \\ 0 & 0 & 0 & 1 \\ \end{array} } \right] \times \left[ {\begin{array}{*{20}c} 1 & 0 & 0 & 0 \\ 0 & {\cos \theta } & {\sin \theta } & 0 \\ 0 & { - \sin \theta } & {\cos \theta } & 0 \\ 0 & 0 & 0 & 1 \\ \end{array} } \right] \times \left[ {\begin{array}{*{20}c} {x^{,} } \\ {y^{,} } \\ {z^{,} } \\ 1 \\ \end{array} } \right]$$
  where $\left( {x,y,z} \right)$ represents the new coordinates of the original point, and $\left( {x^{,} ,y^{,} ,z^{,} } \right)$ the coordinates of the original point.
2. The scaling models were generated by the linearly scaling transformation as shown in the following formula:
  2 $$\left[ {\begin{array}{*{20}c} {x_{t} } \\ {y_{t} } \\ {z_{t} } \\ \end{array} } \right] = \left[ {\begin{array}{*{20}c} {1 + \frac{{a \times \left( {z - z_{m} } \right) \times \left( {k - 1} \right)}}{h}} & 0 & 0 \\ 0 & {1 + \frac{{b \times \left( {z - z_{m} } \right) \times \left( {k - 1} \right)}}{h}} & 0 \\ 0 & 0 & {1 + \frac{{\left( {z - z_{m} } \right) \times \left( {k - 1} \right)}}{h}} \\ \end{array} } \right] \times \left[ {\begin{array}{*{20}c} {x - x_{m} } \\ {y - y_{m} } \\ {z - z_{m} } \\ \end{array} } \right];\quad (z > z_{m} )$$
  where $\left( {x_{m} ,y_{m} ,z_{m} } \right)$ represents the coordinates of the geometric center of the aneurismal neck, $\left( {x,y,z} \right)$ the new coordinates of the original point, $\left( {x_{t} ,y_{t} ,z_{t} } \right)$ the coordinates of the scaled transformed point, h the perpendicular height of the aneurysms from the neck plane to the aneurysmal dome, k the scaling factor and a, b, c the scaling coefficients along x, y, z coordinates, respectively.

Details of the scaling method and its theoretical basis have been described in our published article [19]. The ARs of the scaling models were 0.3, 0.5, 0.7, 1.0, 1.3, 1.5, 1.7 and 2.0, respectively. The choice of AR in this study was based on the AR distribution in patient dataset from Beijing Tiantan Hospital and the suggestions of clinicians. The scaling mechanism was developed according to the phenomenon that the expansion rate of aneurysm increased monotonically from the aneurysmal sac neck to the fundus during aneurysm expansion [20]. In all the scaling models, the parent artery and the aneurysmal neck were kept unchanged.

### Numeric modeling, simulation methods, and boundary conditions

**Fig. 1:**
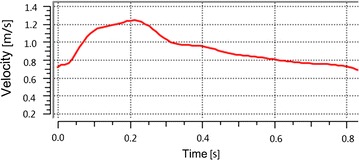
Physiologic flow conditions derived from TCD measurements on a normal subject

To describe the characteristics of the blood flow, we calculated the average Reynolds numbers (Re < 760) of all the original models (the scaled models and their original models have identical parent vessels, we only need to calculate the Re of the original models). The blood in this study was assumed to be an incompressible Newtonian fluid in laminar flow, with a density of 1050 kg/m^3^ and a dynamic viscosity of 0.0035 Pa [19]. Besides, the vessel was modeled as made of a hyperelastic and isotropic material with a density of 1150 kg/m^3^, a Poisson’s ratio of 0.45 and Young’s modulus of 1.2 MPa [19]. The blood flow varied with the cardiac cycle, so pulsating flow condition was imposed on the inlet boundary, as shown below (Fig. 1). A zero pressure gradient along the flow direction was applied at the outlets. No-slip flow boundary condition was imposed on the artery and aneurysmal wall. For a longitudinal comparison, the same boundary condition was applied to all the scaled models (including their original models).

### Computational tools

Commercial software Workbench 13.0 (ANSYS Inc, Canonsburg, PA, USA) was used to generate meshes. The fluid model was constructed with unstructured tetrahedral shell elements and solid model hexahedral shell elements. Moreover, the fluid–solid interface meshes near the arterial wall were prismatic shell elements. Meanwhile, the mesh numbers were set to be 800000 on the basis of a grid independency test, where EL converged to a constant value. In this work, the mesh numbers in all the scaled IAs models ranged from 800,000 to 1,200,000.

In addition, FSI simulation and calculation were performed in Workbench 13.0 (ANSYS Inc, Canonsburg, PA, USA) based on the finite volume method. We simulated a total of 3 cycles to ensure that the initial transients vanish. The workstation of Workbench software is run with two Intel(R) Xeon(R) processors of 2.40 GHz, a Windows operating system (Version 7)and a RAM of 24.0 GB. The computing time was approximately 4.6 CPU hours for each IA model.

### Energy loss calculation

**Fig. 2:**
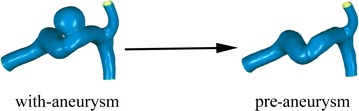
Creating pre-aneurysm. Creating pre-aneurysm by removing the aneurysm from parent vessel artificially

**Fig. 3:**
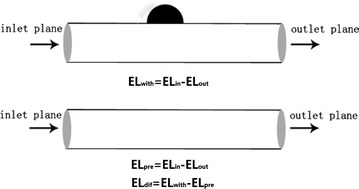
The method of EL calculation. EL is calculated from kinetic energy and total pressure in the artery domain inlet and outlet, and EL of aneurysms in this study is described by the EL difference between the “with-aneurysm” and “pre-aneurysm” conditions

Takao et al. [16] proposed a hemodynamic parameter EL for evaluating the rupture risk of IA. Generally, the main factors for EL are flow separation, turbulence, surface friction, and flow attachment when the fluid passes through complex units. The flow patterns of aneurysm are very complex, accompanied by jet flow, swirling, separating flows, and flow attachment. In order to explore the changes in EL due to the formation of aneurysm, we supposed a pre-aneurysm situation (i.e., the situation occurring before aneurysm formation). The pre-aneurysm state was artificially reconstructed by virtually removing the aneurysm from its parent vessel with Geomagic 12.0 visualization software (Raindrop Geomagic, Durham, USA) (Fig. 2). The EL of aneurysms in this paper was described by the EL difference between the “with-aneurysm” and “pre-aneurysm” conditions (Fig. 3). EL is the EL per unit volume, and it comprehensively represents energy expenditure due to viscous friction and whirlpool formation. The EL was calculated by Eq. 3.

3 $${\text{EL}}_{\text{dif}} = (EL_{{w{\text{ith}}}} - EL_{\text{pre}} )/V_{A}$$

Here $EL_{with}$ represents the energy loss of with-aneurysm condition, $EL_{\text{pre}}$ is the energy loss of non-aneurysm condition, and $V_{A}$ is the aneurysm volume.

EL is expressed by Bernoulli’s equation:

4 $$EL = \nu_{i} A_{in} \, (P_{i} + \frac{1}{2}\rho \upsilon_{i}^{2} + \rho gh_{i} ) - \nu_{o} A_{ot} (P_{o} + \frac{1}{2}\rho \upsilon_{o}^{2} + pgh_{o} )$$

Here P (Pa) represents the total pressure of the inlet or outlet test plane, v (m/s) is the average velocity of the inlet or outlet test plane, g (m/s2) stands for the gravitational acceleration, and subscripts $i$ and o indicate the inlet and outlet of artery, respectively; $A_{in}$ and $A_{ot}$ represent the areas of the test plane at the inlet and outlet of the measurement region, respectively.

The average of the instantaneous EL at each pulse was calculated:

5 $$\overline{EL} = \frac{{\sum\nolimits_{{{\text{i}} = 0}}^{\text{n}} {EL_{i} } }}{n}$$

where n (=800) is the number of time-steps at each pulse.

In addition, the simulations of the pre-aneurysm cases were performed with the same boundary condition as those of the with-aneurysm condition.

## Result

By means of hemodynamics simulation calculation, the hemodynamic longitudinal outcomes of simulated scaling models of IA were obtained.

### Flow pattern

**Fig. 4:**
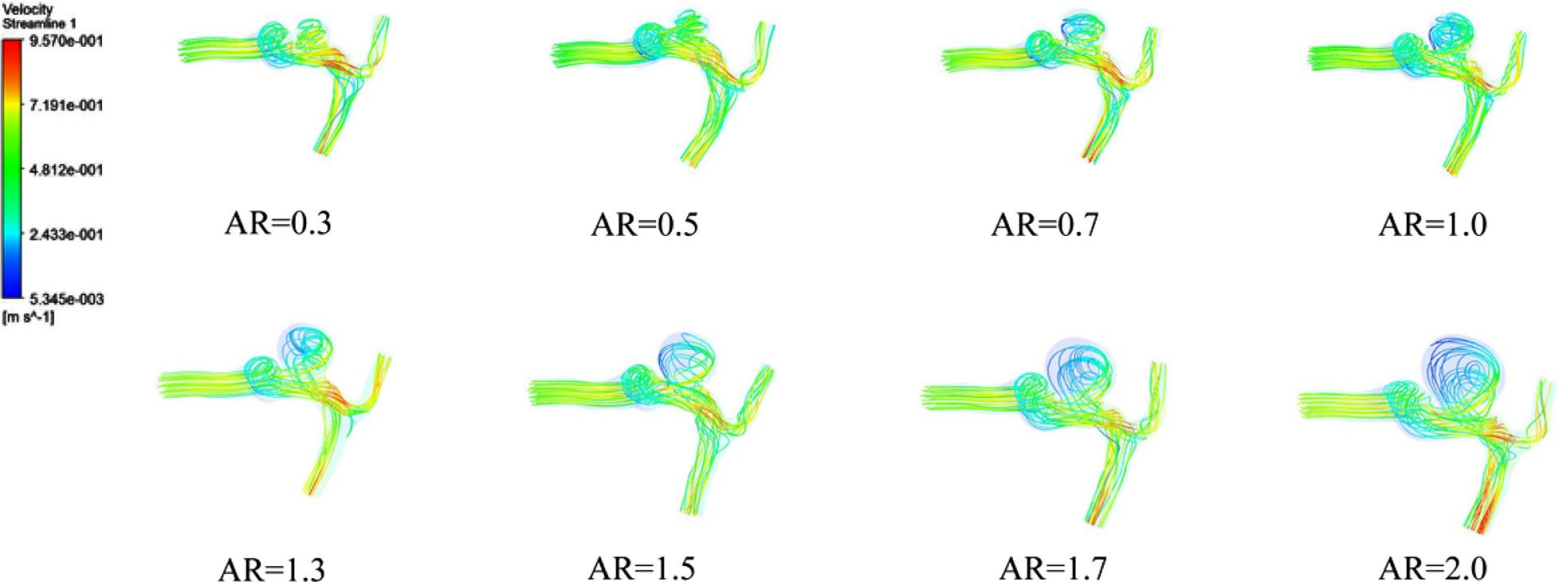
Blood flow streamlines within the aneurysm models at peak systole. The image described the flow velocity, quantity, and vortex in all the scaling models. Obvious variations could be observed with increasing AR

**Fig. 5:**
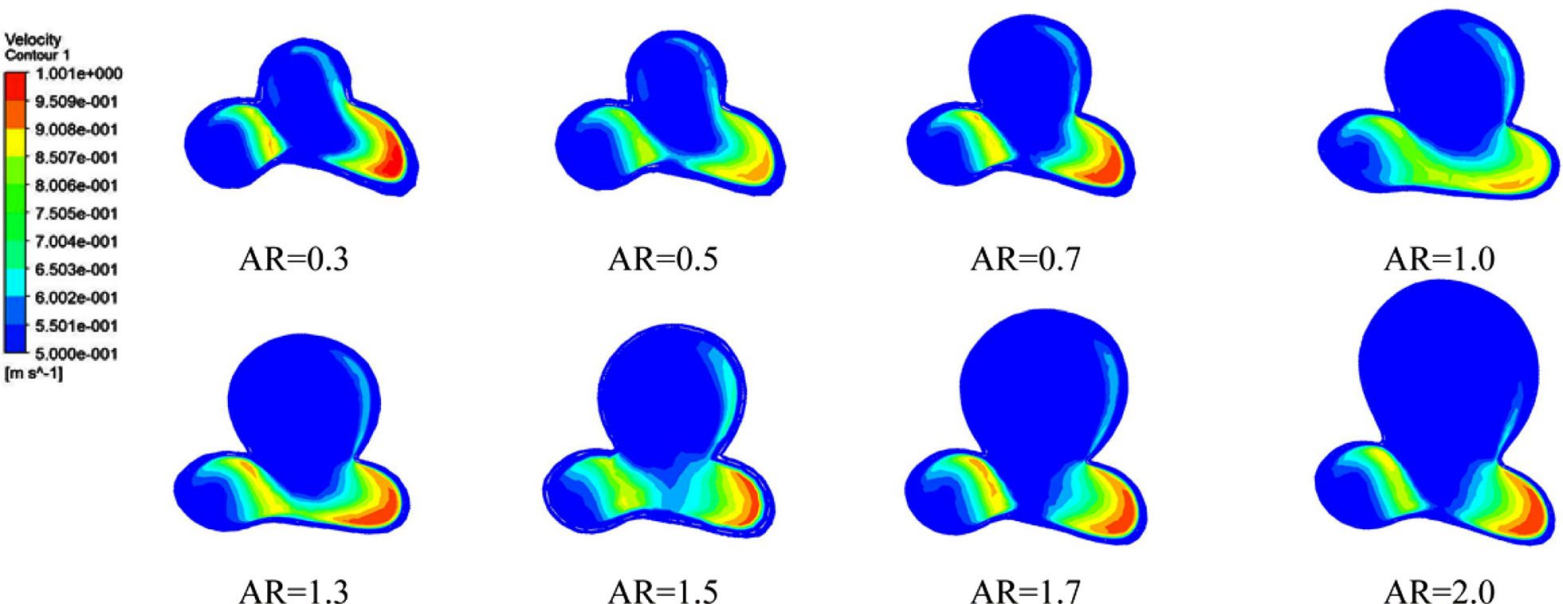
The manifestations of inflow jet in aneurysm models with varied ARs. The inflow jet became narrower as AR increased especially when it exceeded 1.5

The flow pattern of every scaling IA models was constructed. Figure 4 shows the blood streamlines within the scaled IA models at peak systole. We compared the flow patterns of scaled IA models with different ARs. We found that as AR increasing, flow patterns became more complex, with swirling vortex-like streams around the center of aneurysmal cavities (secondary flows). Furthermore, the blood circulated over longer tracks within the IA with higher AR. Especially, in high AR aneurysms (AR > 1.5), the velocity was significantly decreased within aneurysm. IAs with AR < 1.5 were likely to have a single recirculation zone or vortex structure. In the IA cases, there was direct flow interaction at the aneurysm surface near the neck area, resulting in the distribution of high-speed flow into the aneurysm. The zones of the flow jet became narrower as AR was increased (Fig. 5). On the other hand, we also found that IAs with AR > 1.5 tended to have multiple vortex structure, slow flow, and concentrated impingement.

### Energy loss

**Fig. 6:**
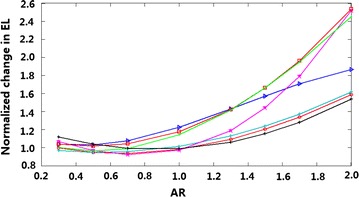
The change of AR to EL. The EL curve showed a clear trend that high AR aneurysms (AR > 1.5) led to drastically higher EL

The EL of every scaled IA models was calculated. In order to better analyze and visualize the longitudinal variation of EL of the simulating IA scaling models, all the resulting EL values were normalized by setting the minimum EL as the reference point. This normalized quantity was referred to as the “normalized change in EL”. The normalized change in EL versus AR was shown in Fig. 6. For each IA model, if the AR was higher, the EL was higher, and high AR aneurysms (AR > 1.5) were found to possess a drastically great EL. IAs with high ARs had swirling, jet flows, and separating flows, and so forth, which were deemed as the main causes of EL. Moreover, the blood circulated over a longer track within the IA with AR > 1.5. Blood had a long turnover time in aneurysm, probably resulting in the consumption of greater energy due to the wear and tear on the aneurysmal wall surface. Flow separation led to changes in velocity. Variation in inflow velocities also presented the change of EL.

## Discussion

In this paper, we proposed a novel method to explore the longitudinal changes of the hemodynamics flow pattern and energy loss (EL) of intracranial aneurysm (IA), a parameter named aneurysm ratio (AR) was acted as a scaling law to create a series of longitudinal models of the original IA model to characterize the longitudinal changes of intracranial aneurysm (IA) hemodynamics flow pattern and energy loss (EL). The advantages of the method are its ability to allow determination of the longitudinal characteristics of the IA, while keeping the parent artery invariant. So the method is especially attractive for eliminating the influence of different parent artery. The experimental results show that EL has a strong correlation with AR. The EL was a potential parameter predicting future rupture of unruptured aneurysms.

The longitudinal hemodynamic outcomes of simulated IA scaling models indicated that the status parameter AR significantly affected the flow pattern and EL. Previously paper published by our team’s research [19] and clinical studies [11, 21] have confirmed that AR had an important effect on IA hemodynamics and might be a useful parameter for predicting IA rupture risk, suggesting an increasing risk of rupture with increasing AR. Meanwhile, our results demonstrated that the EL was significantly increased with AR > 1.5. On the basis of above, one can draw a conclusion that the EL during the growth of the unruptured aneurysms increased sharply is regarded as an increased risk of rupture of an intracranial aneurysm. EL might also be used to predict rupture risk of aneurysms. If the EL during the growth of the unruptured aneurysms increased sharply, we strongly recommend an intervention.

However, there were two limitations in this study. One was that a representative normal subject waveform was used for our simulations. The waveform did not change according to different patients, but also within one patient, depending on his/her physical and mental activities [22]. Nonetheless, previous studies indicated that the CFD solution was insensitive to changes in the waveform [23, 24]. The second limitation was that Newtonian flow was assumed in the simplified models, which would also affect our quantitative CFD results to some extent.

## Conclusion

We proposed a longitudinal model-based scaling method to detect the changes of flow pattern and energy loss in response to altered AR. We found that when AR exceeded 1.5, flow patterns became more complex and EL had a drastic increase. Our findings assessed the effect of the EL on hemodynamics and its implications for rupture prediction. If the EL during the growth of the unruptured aneurysms increased sharply, we strongly recommend an intervention. Meanwhile, these results also provided hemodynamic support for the existing correlation of EL with rupture risk.

## Abbreviations

EL: energy loss
AR: aneurysm ratio
IA: intracranial aneurysm
FSI: fluid-structure interaction
3DRA: 3-D rotational angiography
